# Results of a Web-Based Survey on 2565 Greek Migraine Patients in 2023: Demographic Data, Imposed Burden and Satisfaction to Acute and Prophylactic Treatments in the Era of New Treatment Options

**DOI:** 10.3390/jcm13102768

**Published:** 2024-05-08

**Authors:** Emmanouil V. Dermitzakis, Andreas A. Argyriou, Konstantinos Bilias, Evangelia Barmpa, Sofia Liapi, Dimitrios Rikos, Georgia Xiromerisiou, Panagiotis Soldatos, Michail Vikelis

**Affiliations:** 1 Euromedica General Clinic, 54645 Thessaloniki, Greece; 2 Headache Outpatient Clinic, Department of Neurology, Agios Andreas State General Hospital of Patras, 26335 Patras, Greece; andargyriou@yahoo.gr; 3 Greek Society of Migraine and Headache Patients, 11743 Athens, Greece; kbilias@hotmail.com (K.B.); liabarmpa@gmail.com (E.B.);; 4 404 Military Hospital, 41222 Larissa, Greece; drikos@hotmail.com; 5 Department of Neurology, University Hospital of Larissa, University of Thessaly, 41110 Larissa, Greece; georgiaxiromerisiou@gmail.com; 6 Independent Researcher, 24100 Kalamata, Greece; soldatosp@gmail.com; 7 Headache Clinic, Mediterraneo Hospital, 16675 Athens, Greece; mvikelis@headaches.gr

**Keywords:** migraine, web-based survey, Greece, epidemiology, burden, symptomatic treatment, preventive treatment, patients’ attitudes and satisfaction

## Abstract

**Objective:** The Greek Society of Migraine and Headache Patients conducted its third in-line population web-based survey in 2023 to ascertain if the burden of the disease and the patients’ satisfaction with conventional and novel migraine therapies are changing compared to our previous findings from 2018 and 2020. **Methods:** The sampling process was based on a random call to participants to reply to a specific migraine-focused self-administered questionnaire, including 83 questions in Greek, which was distributed nationwide through the online research software SurveyMonkey. **Results:** We eventually enrolled 2565 patients, the majority of which were females. Our findings clearly demonstrate that migraine is still a burdensome condition. The degree of its impact on all aspects of productivity depends on the monthly frequency of migraine and the response rates to acute and prophylactic treatments. A total of 1029 (42.4%) of the patients had visited the emergency room mainly for unresponsiveness to acute treatments or aura-related symptoms. Triptans seem to be partly effective as acute therapies. OnabotulinumtoxinA seems to be effective for almost half of chronic migraine patients (43.9%) to report adequate satisfaction with this treatment (27.8% were “fairly happy”, 10.6% were “very happy”, and 5.5% were “extremely happy”). Due to their high rates of preventative effectiveness, most respondents treated with anti-CGRP Mabs expressed their optimism concerning their future while living with their migraine (88.25%), as well as towards further improvements in their quality of life (82.8%) status, mostly with fremanezumab. **Conclusions:** The patients recognize the usefulness of anti-CGRP Mabs in migraine prevention and consequently seem to be more optimistic than before about living with migraine. Considering the market change that is anticipated with the use of gepants and ditans, larger longitudinal population-based studies are warranted to further explore if the new era of migraine therapeutics might further lessen the burden of the disease.

## 1. Introduction

Although less frequent than tension-type headaches, migraine, with a prevalence of about 15%, is much more disabling, representing the second most disabling condition among all and the seventh most common disease worldwide, thus imposing a significant burden on all age populations [1]. Particularly, women in their most productive age range of 20–50 years are prone to living with a quite diminished quality of life due to their migraines [2].

Particularly, chronic migraine (CM), defined as more than 15 monthly headache days (MHD), of which at least 8 are of the migrainous type, for more than a trimester [3], might pose an excessive burden to patients because of a longer average duration of headache, elevated pain intensity and increased pain-related comorbidities compared to episodic migraine (EM) [4,5]. Health care resource utilization, and direct and indirect health costs are also higher in CM than in EM [6,7]. Depending on the MHD experienced by patients, EM is defined as headaches accounting for less than 15 days monthly, with 4–8 days monthly sub-classified as low-frequency and 8–14 MHD as high-frequency episodic migraine (HFEM) [3].

Hence, a quite complex framework, considering the multivariate aspects of migraine and other primary headaches, should be implemented in order to define strategies for improved pharmacological and non-pharmacological management in order to reduce the relevant burden of primary headaches. Nonetheless, not only the involvement of headache experts is needed in order to foster the advancement of migraine and headache science, but patients’ opinions and preferences should also be heard. Thus far, there are several publications of results from observational studies implementing the use of internet or telephone-led surveys, which provided data on the demographic and epidemiological data of migraineurs, as well as on the imposed disability and burden of the disease [8,9,10,11].

In addition to the findings of these population-based studies, additional knowledge is gained by the findings of surveys conducted by associations of patients with migraine in order to directly ascertain the impact of the disease on their members or followers. In 2018, the Greek non-profit Society of Migraine and Headache Patients (GSMHP), which belongs to the Pain Alliance Europe and European Migraine & Headache Alliance, released the results of a web-based survey in a sample of 1091 patients with migraine to provide insights on the prevalence and burden of the disease on a national Greek level to raise public and state awareness. This study concluded that migraine at that time was both an underdiagnosed and undertreated disease, whereas patients had little expectations from the available therapies due to their modest efficacy and poor safety [12]. In 2020, while in the middle of the COVID-19 pandemic, GSMHP conducted a web-based survey on 2015 migraine patients with similar objectives as the previous publication in order to determine if any differences occurred. These findings showed insignificant differences in patients’ attitudes, compared to those obtained in the 2018 pre-COVID-19 survey, as it was evident that migraine posed a comparably significant burden and impacted patients’ quality of life. Moreover, it was evident that about two thirds of surveyed patients expressed quite low satisfaction with the available prophylactic medications as a consequence of poor efficacy or intolerability [13,14].

We performed the current population web-based survey in 2023 to ascertain if the burden of the disease as well as the patients’ satisfaction with conventional and novel migraine acute and preventative therapies are changing compared to our previous findings obtained in 2018 and 2020.

## 2. Methods

Data collection occurred over the period from 1 to 30 July 2023. No formal sample size calculation was carried out, and the sampling process was based on a random call to participants to reply to a specific migraine focused self-administered questionnaire, including 83 questions in the Greek language, which was distributed nationwide (in all 13 geographical regions) through the online research software SurveyMonkey.

The same methodology used in the previous studies conducted by GSMHP in 2020 [13,14] was generally employed for conducting the current survey. Briefly, the steering committee of GSMHP sent personal emails to all migraine sufferers who were members of the society, inviting them to participate in the survey, while additional invitations were generally conveyed through GSMHP’s social media accounts (Facebook, Instagram, and Twitter), asking migraine patients who did own a GSMHP membership to also take part in this project. In 2023, GSMHP formally had over 2000 members and 10,500 followers on its social media accounts, including Facebook, Instagram, and Twitter.

This study consisted of two phases. First was the interview, where adult participants of both genders were asked to provide their demographic, socio-economic, and headache clinical data, and then answered key clinical questions to check if the current diagnostic criteria for a definite migraine diagnosis were fulfilled [3]. Finally, patients were asked to clarify if they have a definite diagnosis of migraine established by a physician or experience clinical symptoms resembling migraine but have not been formally diagnosed by a physician. Only participants with a definite diagnosis of migraine with/without aura were asked to proceed to the second phase (the online survey), whereas all others were instructed to stop filling-out the survey and withdraw. During the online survey phase, participants had to provide data concerning the severity and effects of migraine on patients’ QOL and daily living activities (working/societal), as well as the patients’ reported satisfaction with the currently available preventive and symptomatic anti-migraine medications.

The research working group consisted of two members of GSMHP with qualifications and experience in methodology, techniques and tools for conducting scientific research, and four headache specialists who assessed and analyzed the extracted data from this survey. This study was performed in accordance with the principles of the Declaration of Helsinki. A mandatory consent question was included at the beginning of the web-based survey and the participants were informed of the purpose of the survey, how the data would be used, that the data are anonymous, and that they had to read and agree. Approval to conduct this survey was obtained from the Ethics Committee of Euromedica General Clinic, Thessaloniki, Greece.

### Statistical Analysis

Descriptive statistics were performed for categorical and continuous variables, depending on their nature. For non-parametric comparisons of two samples, the Mann–Whitney U test was used, whereas the Chi-square one-sample test and Chi-square with Yates-corrected *p*-value computed results in the comparison of proportions. All tests were performed using SPSS for Windows (release 27.0; SPSS Inc., Chicago, IL, USA). Significance was set at the *p* < 0.05 level.

## 3. Results

**Figure 1:**
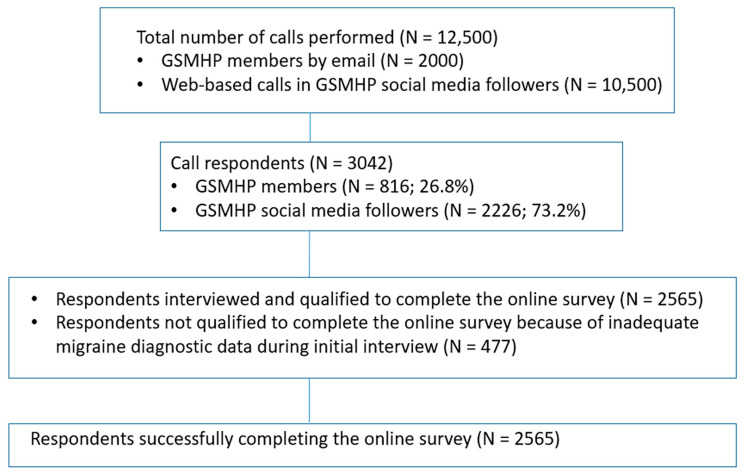
The flow diagram of participants.

After the 12,500 total calls performed (*N* = 2000 to GSMHP members by email and 10,500 web-based calls to GSMHP social media followers), the study eventually enrolled 2565 patients who, after completion of the first diagnostic part of the questionnaire, fulfilled the diagnostic criteria for migraine according to both the ICHD-3 symptom criteria and physician diagnostic criteria. The flow diagram of participants is presented in Figure 1.

### 3.1. Epidemiology and Clinical Characteristics of Surveyed Patients

The study sample consisted of 169 males (7.6%) and 2.369 females (92.4%) (range: 18–64 years). The majority of the patients (73.9%) were between 36 and 55 years old and 62% graduated from university. Comorbidities were common, being mostly clinically manifested as anxiety (29.6%), hypo/hyperthyroidism (24%), irritable bowel syndrome (17.2%), allergies (16%), depression (11.6%), diabetes and/or hypertension (8.3%), and asthma (6.5%).

Among the 2565 participants, 1994 (77.8%) of them already had a formal diagnosis of migraine by a neurologist/headache expert. The majority of participants (*n* = 1934; 75.4%) were classified as having episodic migraine (1–14 MHD), while 24.6% of the patients had CM (at least 15 MHD). The duration of migraine (answers to the question: “*For how many years have you suffered from migraine*?”) was 0–10 years in 20.9%, 11–30 years in 58.6%, and more than 30 years in 20.5% of the participants, whereas 33% reported the onset migraine attacks before adulthood.

The subjective event that was deemed to be associated with the onset of migraine (answers to the question “*Which important event do you think is related to the onset of your migraines?*” (answered by 648 patients) was the death of a loved one in 11.7%, physical/psychological abuse in 12.8%, the onset of menstruation in 13.6%, and pregnancy or childbirth for 14.4% of patients, while about one third of the study sample (33%) did not mention any specific event. A family history of migraine (answers to the question “*Is there a relative of yours who also suffers from migraines?*” was reported by 1821 patients (71.1%), with 34.2% of this subgroup referring to their mother; 12.9% to their father; 17.6% to a sibling; 8.4% to a child of theirs; 16.9% to a grandparent; and 20.7% to an uncle, aunt or cousin (answer to the question “*Which relative of yours suffers from migraine?*”).

**Figure 2:**
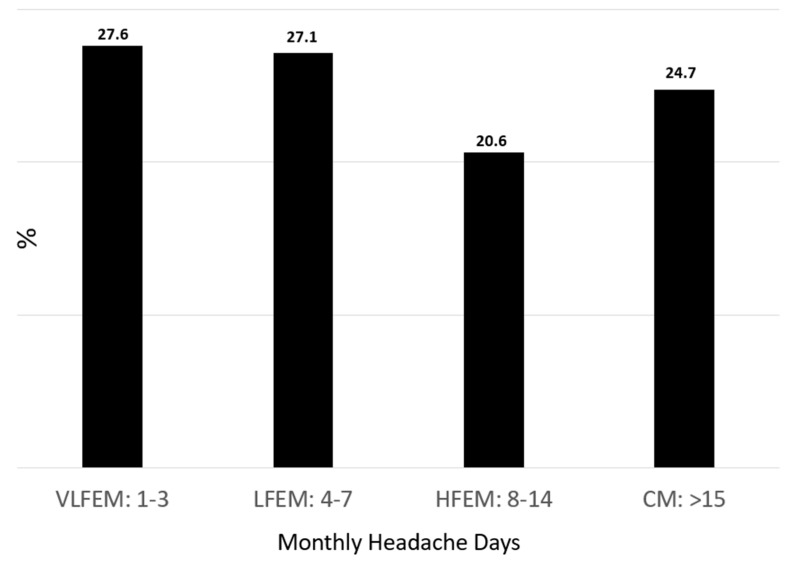
Answers to the question: *“How many days in a month (on average) do you experience headache?*” (abbreviations: VLFEM: very-low-frequency episodic migraine; LFEM: low-frequency episodic migraine; HFEM: high-frequency episodic migraine; CM: chronic migraine).

The answers to the question “*How many days in a month (on average) do you experience any headache symptoms?*” are shown in Figure 2 and demonstrate that about half of our participants (54.7%) had very-low- or low-frequency episodic migraine, while HFEM and CM were also well represented in our sample.

**Figure 3:**
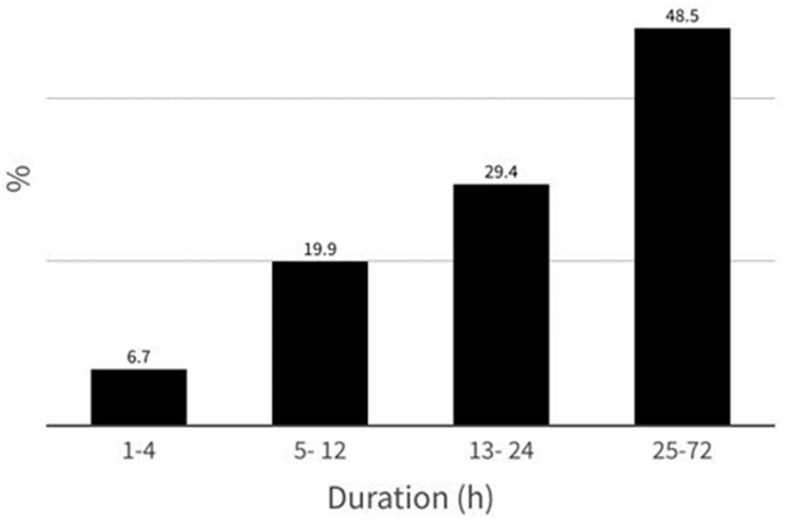
Answers to the question “*How many hours does your migraine attack usually last if you do not take a painkiller or if you take one but it does not work?*”.

Most participants (77.9%) experienced migraine attacks of very long duration, ranging between 13 and 72 h. The answers regarding the duration of migraine are shown in Figure 3.

**Figure 4:**
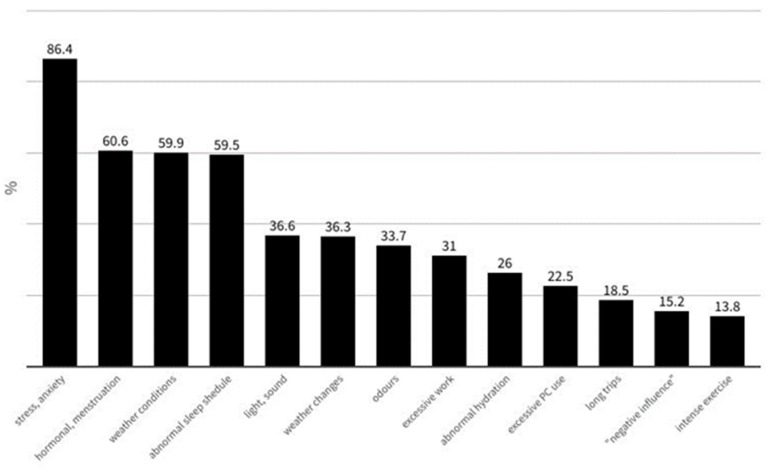
The answers to the question: “*Which factor(s) do you consider to be the main trigger for your migraine?*” (multiple choices).

Triggers of migraine were frequent, with stress/anxiety and menstruation being most commonly reported. The answers to the question “*Which factor(s) do you consider to be the main trigger for your migraine?*” are shown in Figure 4.

**Figure 5:**
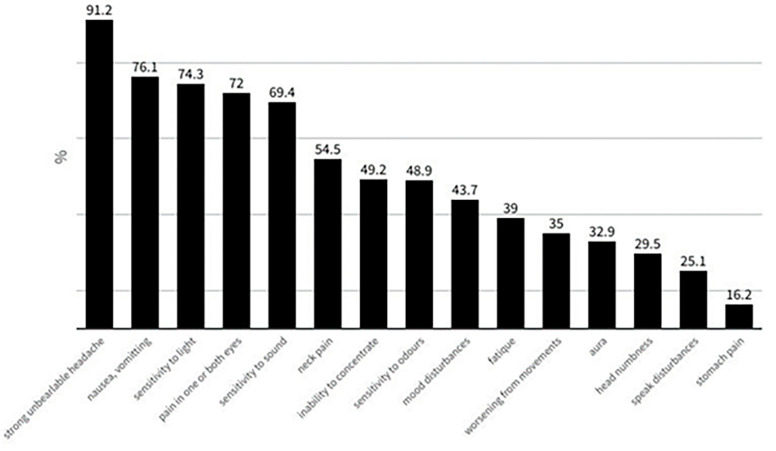
The answers to the question: “*Which symptom(s) do you experience during a migraine attack?*” (multiple choice).

Apart from headache, several other accompanying symptoms were recorded, including nausea/vomiting, photo- and phonophobia, as well as neck pain. The answers to the question “*Which symptom(s) do you experience during a migraine attack?*” are shown in Figure 5.

Within the last three years, 1029 (42.4%) patients had visited the emergency room at least once for poor response to acute treatments during migraine attacks or aura-related symptoms, and 297 (12.2%) had been admitted for hospitalization. About half of the patients (*n* = 1161; 45.3%) reported the intake of acute medication for migraine at least 3 days per week if they are not under prophylactic treatment, and 821 (32%) reported six or more painkillers per week for migraine; both conditions are in keeping with headaches related to comorbid medication overuse [3].

#### 3.1.1. Impact on Work

The questions were addressed to full-time-employed patients. To the question “*Approximately how many days per month are you absent from work due to migraine?*”, 35.2% of the responders answered “1–2 days”, 11.7% answered “3–5 days”, 6.9% answered “more than 5 days”, while 46.2% answered “none”. To the question “*how many days per month do you have a reduction in your performance at work due to migraine?*”, 8.7% of the participants answered “none”, 36.6% answered “1–2 days”, 29.2% answered “3–5 days” and 25.5% answered “more than 5 days”. To the question “*Which of the following has happened to you because of your migraine…?*” the answer “I reduced my working hours” was given by 12.3% of participants; “I lost my job” by 5.3%; “I changed the subject of my work” by 6.7%; and “I took a long leave from my work” by 2.3%. Nevertheless, the majority of participants (73.5%) answered none of the above in reply to the latter question.

#### 3.1.2. Impact on Family and Social Life

To the question “*Approximately how many days per month are you not able to meet your family obligations due to migraine (if you do not take preventive treatment)?*”, 37.3% of the participants answered “1–2 days”, 27% “3–6 days”, 9.7% “7–10 days”, and 9.2% “more than 10 days”, while 16.7% clarified that they do not miss family obligations due to their migraine. To the question “*Approximately how many days per month are you unable to meet your social obligations due to migraine (if you don’t take preventive treatment)?*”, 39.4% of the participants answered “1–2 days”, 27.2% “3–6 days”, 9.9% “7–10 days”, 9.7% “more than 10 days”, and 13.8% reported that they do not miss social obligations due to migraine.

#### 3.1.3. Emotional Impact

**Figure 6:**
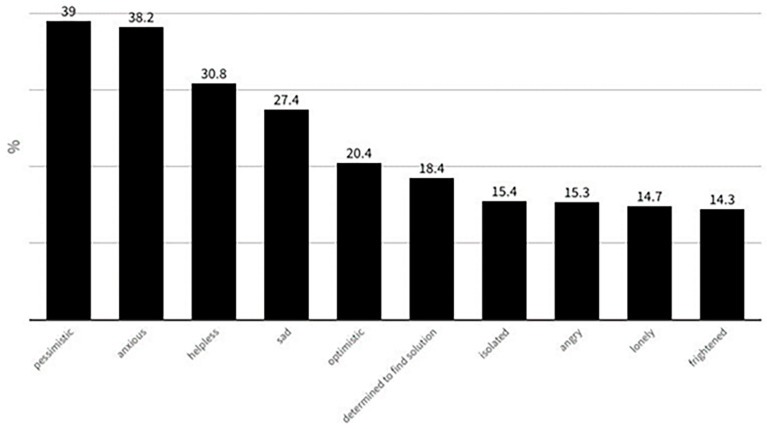
The replies to multiple-choice question: “*Please, choose up to 4 words from the following to describe how you feel about your life with migraine*”.

The replies to multiple-choice question “choose up to 4 words from the following to describe how you feel about your life with migraine” is shown in Figure 6.

### 3.2. Treatment

#### 3.2.1. Acute Migraine Treatment

**Figure 7:**
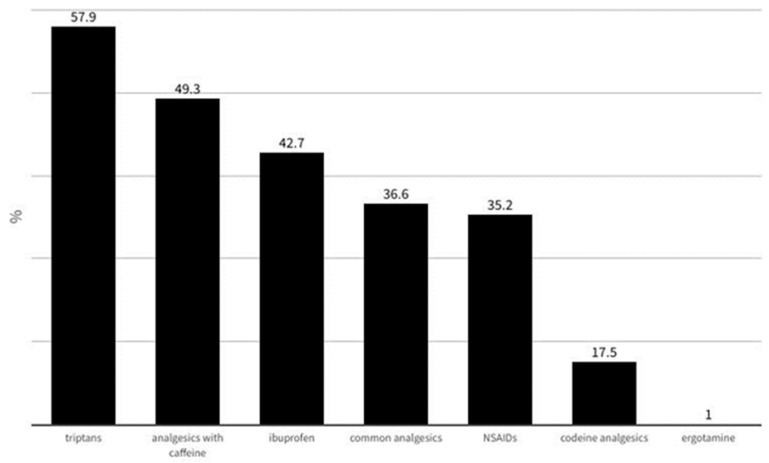
The answers to the multiple-choice question “*During migraine attacks, what symptomatic treatment do you receive?*”.

From the overall sample, 2425 (94.5%) participants systematically used symptomatic treatment (question “*Do you receive symptomatic treatment (painkillers) during a crisis?*”). The answers to the multiple-choice question “*During migraine attacks, what symptomatic treatment do you receive*?” are shown in Figure 7.

When asked, “*How long after the onset of a migraine crisis do you receive your symptomatic treatment*?”, 1115 (45.8%) patients answered “immediately”; 581 (23.9%) “up to 1 h”; 459 (18.9%) “1–2 h”; and 278 (11.4%) “over 2 h”. About half (55.9%) of those who did not take immediate symptomatic treatment answered that they tried to avoid medication overuse. The additional use of Complementary and Alternative Medicine (CAM) as acute treatment of migraine attacks was reported by 2398 (93.4%) patients. The answers to the multiple-choice question “*What alternative symptomatic treatment do you use to relieve your migraine attack in case you do not use conventional medical treatment?*” are “sleep/isolation in a dark room” in 69.8%; “massage” in 33.9%; “cold/hot compresses” in 27.9%; “yoga/meditation” in 4.3%; and “sex/orgasm” in 3.9%.

##### Simple Analgesics, Ibuprofen and Combination of Paracetamol with Caffeine or Combination of Paracetamol, Acetylsalicylic Acid and Caffeine

A total of 1731 (67.4%) patients reported systematic consumption of various simple analgesics, such as paracetamol and acetylsalicylic acid for acute migraine pain treatment.

In addition, 1426 (55.6%) patients reported systematic consumption of ibuprofen for acute migraine pain treatment.

Finally, 1647 (64.2%) patients reported using some combination of paracetamol and caffeine, while about half of participants (*n* = 1303; 50.8%) reported using some combination of paracetamol, acetylsalicylic acid, and caffeine.

The ratings of patients’ satisfaction with simple analgesics, ibuprofen, paracetamol/caffeine combination, or paracetamol/acetylsalicylic acid/caffeine combination are shown in Table 1. The ratings are based on the replies to the question “*How happy are you with the way you are treating migraine with either of the acute treatments*?”

##### Triptans

Triptans were used by 1485 (57.9%) patients for acute migraine pain. A total of 1063 (71.6% of triptans users) patients used rizatriptan; 918 (61.8%) sumatriptan; 740 (49.8%) eletriptan; 292 (19.6%) almotriptan; and 282 (18.9%) naratriptan. Frovatriptan is no longer available in Greece. The ratings of patients’ satisfaction with every triptan medication are shown in Table 2.

#### 3.2.2. Prophylactic Anti-Migraine Treatments

Among the surveyed participants, about half of them (*n* = 1152; 58%) had received various prophylactic treatments for migraine at least once.

##### Antiepileptics (Topiramate and/or Valproic Acid)

A total of 683 (59.2%) of the responders had received antiepileptics for their migraine prophylaxis at least once, either topiramate or valproic acid. For topiramate, just 7.1% were “very happy”, and 5% were “extremely happy” (Table 3). There were comparable rates of satisfaction for valproic acid as just 4.8% were “very happy”, and 0.6% were “extremely happy”. Most participants treated in the past with antiepileptics (*n* = 550; 80.5%) answered positively to the question: “*Did you stop taking antiepileptics for some reason?*”. Of those, 254 (46.2%) stopped due to ineffectiveness and 273 (49.6%) because of side effects including weight loss or gain, agitation, dizziness, somnolence, numbness, and clumsiness or unsteadiness.

##### Antidepressants

In total, 608 (52.8%) participants had been treated with antidepressants as a prophylactic treatment for migraine at least once with SSRIs or SNRIs (duloxetine, venlafaxine, citalopram) or with tricyclics (amitriptyline). However, just 32.2% of patients reported some degree of satisfaction with all kinds of SSRIs/SNRIs or tricyclics (Table 3). Amitriptyline demonstrated rates of satisfaction quite similar to those of venlafaxine (32.8%), with 20.1% of patients being “fairly happy”, 6.4% “very happy”, and 6.3% “extremely happy”. Lower rates of satisfaction (29.5%) were reported for citalopram and for duloxetine (26.7%).

To the question “*have you stopped antidepressants for some reason*?”, 382 (62.9%) patients answered positively. Among them, 49.8% stopped the antidepressants due to ineffectiveness, and 47.6% due to side effects including weight gain, agitation, dizziness, somnolence, and indigestion or stomach aches.

##### Beta Blockers (Propranolol/Metoprolol) or Calcium Channel Blockers

Prophylactic treatments with either beta blockers (propranolol or metoprolol) or calcium channel blockers (flunarizine) were commenced in 321 (27.8%) and 442 (38.4%) patients, respectively. For propranolol, 4.3% were “very happy” and 4.3% were “extremely happy. For metoprolol 6.5% were “very happy” and 3.9% were “extremely happy”. The vast majority (88.9%) of beta blockers-treated patients discontinued treatment due to side effects, such as bradycardia, irritability, and mood disorders, while ineffectiveness (48.3%) and weight gain (51.4%) were the main causes of flunarizine discontinuation.

##### OnabotulinumtoxinA (BoNTA)

In total, 257 (22.3%) patients answered that they had BoNTA injections for CM prophylaxis (of a total of 604 patients who had headaches more than 14 days/month). A total of 208 (81.3%) of them reported that their treatment was performed with the approved site-fixed dose-fixed PREEMPT protocol by a trained neurologist [15]. Most BoNTA-treated patients (65.4%) had received at least three quarterly cycles of injections (with 6.4% having ≥12 cycles of injections); 18.1% had received one cycle of injection; and 16.5% two cycles. The remaining 49 patients received BoNTA by dermatologists or ENT physicians at arbitrary and unapproved injection sites, i.e., just in the forehead, and dosages. To the question, “*How happy are you with the effect of BoNTA treatment in migraine prophylaxis?*”, 43.9% of patients reported adequate satisfaction with this treatment (Table 3). To the question, “*Why did you stop your treatment with BoNTA*?”, 54.1% responded “due to ineffectiveness”, 22.1% “due to the out-of-pocket cost”, and 7.8% “due to side effects”, mainly neck pain.

##### Anti-CGRP/Anti-CGRPr Monoclonal Antibodies

A total of 315 patients were receiving anti-calcitonin gene-related peptide (CGRP) monoclonal antibodies (anti-CGRP/anti-CGRPr MAbs) because, in Greece, fremanezumab was approved for reimbursement by the Greek National Health System (NHS) and social services in July 2021, erenumab followed in February 2022, and galcanezumab in January 2023. From the question “*For how many months have/did you receive anti-CGRP treatment?*”, it was evident that anti-CGRPs were given to participants for an average of 15 months (range 1–25 months). Fremanezumab was given to 216 (68.6% of anti-CGRP MAbs users) patients, 48 (15.2%) were treated with erenumab, and 51 (16.2%) with galcanezumab. Notably, 40 patients switched from one to another anti-CGRP Mabs, mainly to fremanezumab from erenumab due to the low efficacy or side effects of the latter monoclonal antibody targeting the CGRP receptor. The patients’ satisfaction with these treatments was perceived as high (36.8%) or very high (46%) by 82.8% of respondents (Table 3). The question “*Have you stopped treatment with monoclonal antibodies for any reason?*” was answered negatively by 277/315 (87.5%), and positively by 38 (12.1%) patients; 30 of them (78.9%) reported “due to lack of effectiveness”, 7 (18.5%) “due to high cost”, and 1 (2.6%) “due to side effects”, i.e., constipation after treatment with erenumab. As a result, 278/315 (88.25%) respondents, under anti-CGRP preventative therapy, expressed a high or very high level of optimism concerning their future while living with their migraine. Likewise, the patients’ perception of improvements in their quality of life, mostly with fremanezumab, was perceived as high (36.8%) or very high (46%) from 82.8% by respondents.

### 3.3. Associations

Participants with CM clearly prioritized effectiveness over safety in medications prescribed for their migraine prophylaxis compared to patients with episodic migraine (odds ratio (*OR*) = 4.7; *p* = 0.01), while significant differences emerged between CM and EM patients in the perception of threshold for adequate satisfaction in a preventative migraine treatment, where CM patients reported that a reduction of at least 50% or 75% (*OR* = 6.1; *p* < 0.001) in their mean headache days (MHD) is needed, while EM patients reported that even a 30% MHD reduction is clinically important (*OR* = 2.1; *p* = 0.01). Gender effects were also evident with female patients experiencing three times higher EM (*OR* = 2.9, *p* < 0.001) and five times higher CM (*OR* = 5.1, *p* < 0.001) compared to male patients. CM patients had a much higher burden (*OR* = 6.2, *p* < 0.001) from the disease than EM patients, with more pronounced effects on their productivity at work and their impact on their family and social life. No other significant differences were documented.

## 4. Discussion

This web-based survey conducted by the GSMHP, targeting advocacy group members and followers after the formal termination of the COVID-19 pandemic, aimed to trace the current burden of the disease in 2023 as well as the patients’ satisfaction with acute and prophylactic migraine therapies in order to ascertain if there are any changing attitudes and trends compared to our previous findings obtained in the surveys held in 2018 and 2020 [13,14]. This appears to be of great interest given the evolving market change occurring in Greece regarding the use of monoclonal antibodies to calcitonin gene-related peptide in the symptomatic and prophylactic treatment of migraine in the past 3 years.

Generally, the updated demographic and epidemiologic findings remained grossly unchanged compared to those obtained in the 2018 pre-COVID-19 and 2020 intra-COVID-19 surveys, thoroughly confirming that, generally, the attitudes of our participants were not significantly affected by the coronavirus pandemic situation despite any potential psychological burden posed by the restrictive measures. The majority of our participants were females (92.4%) at a productive phase of their lives (73.9% were between 36 and 55 years old). These findings are in keeping with the majority of large epidemiological studies in migraineurs showing a clear and strong gender predominance of females over males, as demonstrated from the findings of a relatively recent survey conducted by the European Migraine & Headache Alliance (EMHA) in 3342 patients in Spain, Italy, France, Portugal, Ireland, the United Kingdom, Germany and other countries of the European Union, where it was demonstrated that over 85% of participants were middle-aged females [16]. However, an interesting finding of our current survey was that about one third of participants had their first migraine attack before the age of 18. This finding further demonstrates that migraine is also frequent and potentially disabling before adulthood [17], but also reflects the perennial nature of migraine, which likely leads to transformation and chronification in about 3% of migraineurs [18,19].

Regarding the impact of migraine on patients’ ability to work, the majority of respondents stated that they experienced occupational effects with a loss of working days (53%) or reduced productivity at work due to migraine (91.3%). These effects were at various degrees, with most respondents stating that they lose 1–2 working days per month (35.2%) and have reduced productivity at work for 1–2 days per month (36.6%). These trends were comparable with those obtained in our previous 2020 survey, in which the corresponding percentages were 35.2% and 38.03% for 1–2 lost working days per month and 1–2 days per month with reduced productivity at work due to migraine, respectively [13,14]. The impact of migraine on work also had financial impacts, with reduced monthly income for 26.4% of patients, mostly due to reducing their working hours or the loss of a paid job. Corresponding percentages of participants in the current survey stated that migraine obviously impacted on their family (37.3%) and social life (39.4%) with an inability to accomplish family and social obligations for at least 1–2 days per month. CM patients experienced increased burden from the disease and more pronounced effects on all the latter domains compared to EM patients. These findings further support the widely accepted view that migraine is a highly burdensome condition, with a consistently strong general trend towards worse outcomes and a higher burden or impact with increased headache frequency [20]. However, although EM is generally considered more benign than CM, there is evidence to support that high-frequency EM patients might have a similar degree of disability and migraine-related burden compared with CM patients experiencing 15–23 MHD [21].

One of the striking findings is that, within the last three years, about half of our participants (42.4%) presented to the emergency room at least once for migraine, mainly for uncontrolled migraine attacks or aura. Our results are generally in keeping with previous data on migraine patients’ access to emergency room and eventual hospitalization [22,23].

Regarding the use of acute migraine treatments, it was evident that the overwhelming majority (94.5%) of respondents took symptomatic medications, including simple painkillers, NSAIDS, and combinations of caffeine with simple analgesics and triptans. However, notably, only about half of participants (57.9%) used triptans, mainly rizatriptan and sumatriptan. Another interesting finding was that only up to 18.6% of patients, using triptans, expressed satisfaction with these medications when the intake of triptans occurred within less than 1 h after the onset of a migraine attack, which is in keeping with current guidelines for controlled trials of acute treatment of migraine attacks in adults [24] and current evidence demonstrating that triptan persistence is low, with a lack of efficacy representing the most common reason for discontinuation [25].

Concerning migraine prophylaxis with the use of orally given conventional and unspecific migraine treatments, either antiepileptics, antidepressants, beta blockers or calcium channel blockers, it was determined that about 60% of participants received either of these medications at least once. However, their satisfaction with these treatments was quite low due to inefficacy or safety issues, and the corresponding rates of discontinuation were high, in keeping with current evidence suggesting that conventional anti-migraine prophylactics have a higher likelihood to harm than to help [26,27]. BoNTA CM prophylaxis was received by 22.3% of respondents, and about half of them (43.9%) reported adequate satisfaction with this treatment, with a reduction of 50% or more in MHD. Tellingly, however, the efficacy of BoNTA and the corresponding patients’ satisfaction rates would most likely be higher if we consider that only 81.3% of patients were treated with the approved site-fixed dose-fixed (155-195UI) PREEMPT protocol by a trained neurologist, while only 65.4% had received at least three quarterly cycles of injections. After treatment with at least three BoNTA cycles is generally acknowledged as the correct timepoint to judge in favor or against a meaningful clinical response to BoNTA treatment, which is also in agreement with our published experience [28,29].

A significant number of participants (*n* = 315) were treated with anti-CGRP Mabs, either targeting the CGRP receptor or the ligand. The patients’ satisfaction with these treatments regarding improvement in the quality of life, mostly with fremanezumab, was perceived as high (36.8%) or very high (46%) by 82.8% of respondents. Likewise, the optimism of these patients was high (88.25%). These results support what is already known about the potent efficacy of fremanezumab in migraine prophylaxis settings, as documented in a pooled analysis of clinical trials and by real-world data [30,31,32,33,34]. Safety issues leading to discontinuations were only observed with erenumab, mainly due to constipation.

The most hopeful and outstanding finding in the current survey, compared with the corresponding 2020 findings, is the change in the way migraineurs feel about their lives with migraine in 2023. Only 6.8% described their feelings as “optimistic” in 2020 [13,14], but this percentage is three times higher in 2023 (20.4%). Three years before, only 5.5% of the migraineurs felt determined to find a solution to the burdensome disease they have, while in 2023, this determination was increased by more than three times (18.4%). In 2020, the migraineurs used the negative words “anxious”, “helpless”, “sad”, “angry” more than in 2023 to describe their feelings about their lives with migraine (49.2% vs. 38.2%, 45.1% vs. 30.8%, 33.7% vs. 27.4% and 22.7% vs. 15.3%). Obviously, the use of anti-CGRPs represents a game changer in relation to migraine prophylaxis. Nonetheless, our results show that we have entered a new era for acute and preventive treatment, and this progress motivates patients to be more optimistic about their lives with migraine in comparison to previous years.

Our study has limitations that may impose bias in the interpretation of our findings, including (i) the cross-sectional design with the use of a web-based survey rather than a prospective study design; (ii) the use of a self-administered questionnaire which had face validity but was not subjected to any form of psychometric process; (iii) the potential bias from targeting subjects with diagnosed migraine in the sampling process; and (iv) the fact that we have not used standardized questionnaires to assess the migraine-related burden and its impact on the overall QOL.

## 5. Conclusions

Nonetheless, our survey clearly demonstrated that migraine is a burdensome condition. Its impact on general productivity depends on the frequency of MHD that the patient experiences with migraine, and the degree of response to acute and prophylactic treatments. Triptans seem to partly provide a prompt and clinically meaningful analgesia of migraine pain, and gepants might be a rational treatment option in patients who remain unresponsive or have contraindications to triptans. Anti-CGRP Mabs are effective and safe for migraine prophylaxis. Considering the longitudinal market change that is anticipated with the use of gepants in the prevention of migraine and with the use of ditans as symptomatic treatment, further larger longitudinal population-based studies are warranted to further explore if the new era of migraine therapeutics might lessen the burden of the disease in the future.

## Figures and Tables

**Table 1:** Patients’ satisfaction to unspecific acute symptomatic treatments for migraine attacks.

	“Not Happy at All” *N* (%)	“Modestly Happy” *N* (%)”	“Fairly Happy” *N* (%)	“Very Happy” *N* (%)	“Extremely Happy” *N* (%)
Simple analgesics (*n* = 1731)	730 (42.2)	613 (35.4)	278 (16.1)	68 (3.9)	42 (2.4)
Ibuprofen (*n* = 1426)	312 (21.9)	445 (31.2)	405 (28.4)	171 (12)	93 (6.5)
Paracetamol/caffeine combination (*n* = 1647)	473 (28.7)	591 (35.9)	402 (24.4)	107 (6.5)	74 (4.5)
Paracetamol/acetylsalicylic acid/caffeine combination (*n* = 1303)	326 (25)	420 (32.2)	375 (28.8)	96 (7.4)	86 (5.6)

**Table 2:** Patients’ satisfaction to triptans’ use for the symptomatic treatment of acute migraine.

	“Not Happy at All” *N* (%)	“Modestly Happy” *N* (%)	“Fairly Happy” *N* (%)	“Very Happy” *N* (%)	“Extremely Happy” *N* (%)
Rizatriptan (10 mg, or.disp)	178 (16.7)	214 (20.1)	256 (24.1)	217 (20.4)	198 (18.6)
Sumatriptan (50 and 100 mg, tabl.)	184 (20)	189 (20.6)	238 (25.9)	173 (18.8)	134 (14.6)
Eletriptan (20 and 40 mg, tabl.)	143 (19.3)	116 (15.7)	178 (24.1)	171 (23.1)	132 (17.8)
Naratriptan (2.5 mg, tabl.)	158 (56)	57 (20.2)	47 (16.7)	14 (5)	6 (2.1)
Almotriptan (2.5 mg, tabl.)	158 (54.1)	69 (23.6)	38 (16.4)	7 (2.4)	10 (3.4)

**Table 3:** Patients’ satisfaction to available oral (topiramate/antidepressants) or injectable medications (OnabotulinumtoxinA/anti-CGRP MAbs) for migraine prophylaxis.

	“Not Happy at All” *N* (%)	“Modestly Happy” *N* (%)	“Fairly Happy” *N* (%)	“Very Happy” *N* (%)	“Extremely Happy” *N* (%)
Topiramate, usually 50 mg b.i.d every 12 h (*n* = 683)	275 (40.2)	254 (37.2)	72 (10.5)	48 (7.1)	34 (5)
SSRIs/SNRIs or tricyclics (*n* = 608)	182 (30.0)	228 (37.5)	111 (18.3)	72 (11.8)	15 (2.4)
OnabotulinumtoxinA 155-195UI quarterly given (*n* = 257)	50 (19.4)	94 (36.7)	72 (27.8)	27 (10.6)	14 (5.5)
Fremanezumab 225 mg/pf-syr every 28–30 days (*n* = 216)	8 (3.7)	22 (10.2)	40 (18.5)	60 (27.8)	86 (39.8)
Erenumab 70/140 mg/pf-syr every 28 days (*n* = 48)	6 (12.5)	8 (16.7)	8 (16.7)	16 (33.3)	10 (20.8)
Galcanezumab 120 mg/pf-syr every 28–30 days (*n* = 51)	3 (5.9)	8 (15.7)	11 (21.6)	13 (25.5)	16 (31.3)

## Data Availability

The corresponding author has full control of all primary data and agrees to allow the journal to review our data upon reasonable request. The data that support the findings of this study are available from the corresponding author upon reasonable request.
